# Speed dating for enzymes! Finding the perfect phosphopantetheinyl transferase partner for your polyketide synthase

**DOI:** 10.1186/s12934-021-01734-9

**Published:** 2022-01-10

**Authors:** Tobias Bruun Pedersen, Mikkel Rank Nielsen, Sebastian Birkedal Kristensen, Eva Mie Lang Spedtsberg, Trine Sørensen, Celine Petersen, Jens Muff, Teis Esben Sondergaard, Kåre Lehmann Nielsen, Reinhard Wimmer, Donald Max Gardiner, Jens Laurids Sørensen

**Affiliations:** 1 Department of Chemistry and Bioscience, Aalborg University Esbjerg, Niels Bohrs Vej 8, 6700 Esbjerg, Denmark; 2 Department of Chemistry and Bioscience, Aalborg University Aalborg, Fredrik Bajers Vej 7H, 9220 Aalborg, Denmark; 3 The University of Queensland, 306 Carmody Rd, St Lucia, Brisbane, QLD 4072 Australia

## Abstract

The biosynthetic pathways for the fungal polyketides bikaverin and bostrycoidin, from *Fusarium verticillioides* and *Fusarium solani* respectively, were reconstructed and heterologously expressed in *S. cerevisiae* alongside seven different phosphopantetheinyl transferases (PPTases) from a variety of origins spanning bacterial, yeast and fungal origins. In order to gauge the efficiency of the interaction between the ACP-domains of the polyketide synthases (PKS) and PPTases, each were co-expressed individually and the resulting production of target polyketides were determined after 48 h of growth. In co-expression with both biosynthetic pathways, the PPTase from *Fusarium verticillioides* (FvPPT1) proved most efficient at producing both bikaverin and bostrycoidin, at 1.4 mg/L and 5.9 mg/L respectively. Furthermore, the remaining PPTases showed the ability to interact with both PKS’s, except for a single PKS-PPTase combination. The results indicate that it is possible to boost the production of a target polyketide, simply by utilizing a more optimal PPTase partner, instead of the commonly used PPTases; NpgA, Gsp and Sfp, from *Aspergillus nidulans*, *Brevibacillus brevis* and *Bacillus subtilis* respectively.

**Supplementary Information:**

The online version contains supplementary material available at 10.1186/s12934-021-01734-9.

## Introduction

The chemical group of compounds known as polyketides exhibit an abundance in structural and bioactive diversity, making them some of the foremost interesting compounds for researching natural products, with a plethora of applications ranging from antibiotics, immunosuppressants, anti-cancer to fungicides and perhaps even as a solution to energy storage in the future [1–5]. Polyketides are attractive targets for large-scale production due to their bioactive properties, which can be achieved in their natural host or in heterologous hosts [4, 6]. A common choice for heterologous expression is the baker’s yeast *Saccharomyces cerevisiae*, which however often suffers from low production yields. Consequently, numerous strategies has been applied to increase production, including metabolic engineering to increase flux towards acetyl- and malonyl-CoA [7, 8] and fusion of tailoring enzymes, as we in this paper suggest an additional method for increased production yields [9–11].

**Fig. 1:**
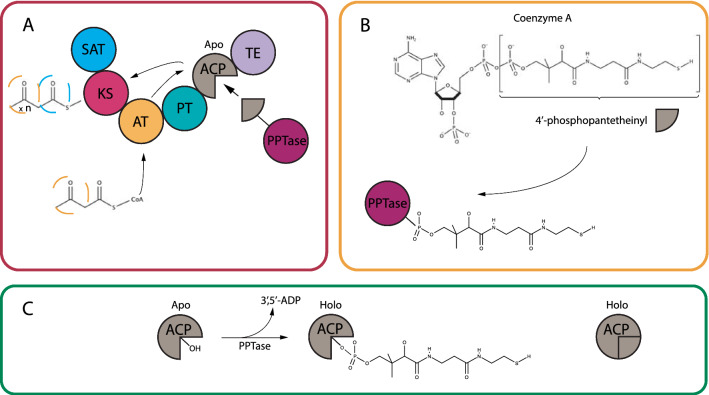
Illustration of the iterative function of a type 1 fungal PKS and interaction with a PPTase. **A** Modular representation of a type 1 fungal PKS containing multiple domains responsible for production of the first polyketide intermediate. **B** Shows the 4′-phosphopantetheinyl moeity taken from Co-enzyme A attached to the PPTase enzyme, enabling the conformational changes to the ACP-domain, as seen in **C** where the inactive apo-form is transformed into the active holo-form

In fungi, biosynthesis of the vast majority of polyketides are initiated by multidomain iterative polyketide synthases (PKS’s), which catalyze condensation of acyl thioesters (primarily acetyl- and malonyl-CoA) [12]. Three conserved domains are found in all iterative PKSs, the β-ketosynthase (KS), acyltransferase (AT) and acyl-carrier protein (ACP), which together constitute a minimal PKS [13]. Chemical diversification is achieved by the actions of additional domains, including the β-ketoreductase (KR), dehydrogenase (DH), enoyl reductase (ER), which catalyze reductions of the polyketide chains before they are released from the PKS [14]. Movement of the growing polyketide chain between the various domains is mediated by the ACP domain, which includes a 4′-phosphopantetheine prosthetic group [15]. This group is activated by a 4′-phosphopantetheinyltransferase (PPTase), of which an adequate homolog is not present in *S. cerevisiae*. Thus, successful heterologous production of polyketides in *S. cerevisiae* requires co-expression of a PPTase. There are two major protein-families of PPTase enzymes (AcpS-type and *Sfp*-type), which can in addition to PKS′s also interact with fatty acid synthases (FAS) and non-ribosomal peptide synthetases (NRPS). The *Sfp-*type are the primary PPTases linked to expression of secondary metabolites in fungi, which is why these are used throughout this work [16]. Through the transfer of a 4′-phosphopantetheinyl moiety from Coenzyme-A, to the ACP domains of PKS′s, FAS′s and PCP (Peptidyl Carrier Protein) domains of NRPS’s, the iterative function of the synthesizing enzymes is maintained. This is accomplished by structural integration of said moiety, transforming the CP-domains from an inactive apo-form to the active holo-form, which must occur for each iteration (Fig. 1) [16–18]. A curiosity of these enzymes, is that they display a large sequence variation, while still maintaining the functionality towards different types of enzymes and perhaps more importantly the conserved ACP-domains they target. Two regions of the Sfp-type PPTase are conserved and denoted ppt-1 and ppt-3, containing specific patterns of amino acid 38–41 and further dissection of this family of PPTase are possible through the sub-motifs of either WxxKEA or FxxKES [16]. The promiscuous nature of these sfp-type PPTases has previously been reported, through the interaction with a broad-range of both PKS’s and NRPS’s [19].

As the 4′-phosphopantetheinylation process is a two-enzyme interaction, between the synthezing enzyme and the PPTase, that regulates the transfer of the 4′-phosphopantetheinyl moiety, we want to investigate whether the PPTase is limiting the function of the PKS resulting in an unwanted bottleneck. The majority of studies with heterologous production of polyketides in *S. cerevisiae* have utilized the same few standard PPTases, Sfp from *Bacillus subtilis* or NpgA from *Aspergillus nidulans* [10, 20], opening the possibility for titer increase through substitution of PPTases. Through a substitutional approach we investigated the standard PPTases alongside five additional PPTase of different origins. One bacterial PPTase, Gsp, from *Brevibacillus brevis*, SpPPT1 from another yeast species *Schizosaccharomyces* pombe to elucidate if a PPTase from a more closely related species is advantageous. Furthermore, three different fungal PPTase were investigated to determine if an evolutionary advantage of PPTases originating from fungi natively expressing these compounds, was evident.

To explore these scenarios, we expressed two biosynthetic pathways native to *Fusarium *spp. resulting in the formation of the polyketides bikaverin and bostrycoidin, in the heterologous host *S. cerevisiae* while combinatorically expressing the seven different *Sfp*-type PPTases simultaneously. The two compounds are of interest due to their variety of applications, such as their bio-active nature [21], pigmentation for usage as dye [22] and furthermore contain a specific structure of interest, namely the quinone-structure. This chemical structure has recently shown potential in energy storage solution and are therefore of interest for down-stream application [5, 23, 24].

An additional experiment combining the phosphopantetheinylating potential of two PPTases (NpgA and FvPPT1) was conducted to elucidate whether previously published work on the stacking effect of PPTases applies to the double-ACP domain containing Fsr1 [25].

## Materials and methods

### Microorganisms

Yeast cloning and heterologous expression were performed utilizing *S. cerevisiae* BY4743 (genotype: MATα, his3Δ1, leu2Δ0, lys2Δ0, met15Δ0, ura3Δ0) ATCC 201390. Cloning and maintaining of plasmids were done in *Escherichia coli* DH5α. *Fusarium solani* FGSC 9596 (*Fusarium vanettenii*) was used as donor of DNA for amplification of *fsr2* [26].

### Enzymes, oligonucleotides and plasmids

All enzymes were purchased from Thermo Fisher Scientific (Waltham, Massachusetts, USA) and oligonucleotides were designed using Primer3Plus software [27] and purchased from Eurofins Genomics (Ebersberg, Germany). Primers designed for cloning are listed in Additional file 1: Table S1 along with restriction enzymes used for digestion. Table of constructed plasmids can be found in Additional file 1: Table S2.

### Cloning, pathway reconstruction and plasmid validation

**Fig. 2:**
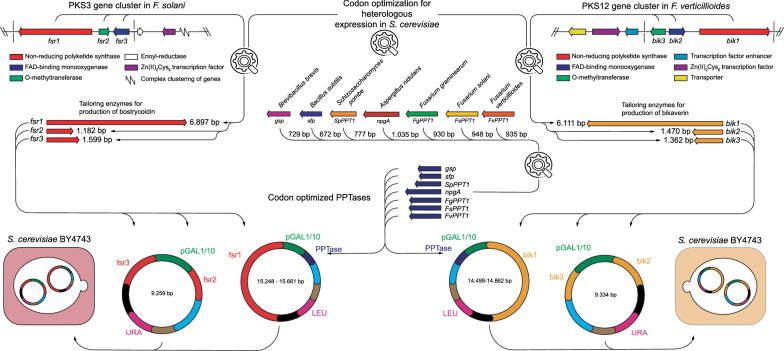
Schematic overview of the biomolecular workflow from target gene clusters, through codon optimization and final cluster reconstruction in plasmids and subsequent transformation into *S. cerevisiae* BY4743

Reconstruction of the bostrycoidin pathway was performed and described previously [9]. An identical workflow was performed for reconstruction of the bikaverin pathway, where all three genes were codon optimized and synthesized. The PPTase genes (Table 1) were codon optimized and synthesized (GenScript Biotech, NJ, USA), before introduction into the pESC-LEU plasmids containing either *fsr1* or *bik1* as visualized in Fig. 2 along with all codon optimized gene sequencing (Additional file 1: Figure S1). To validate the plasmid constructs, all final plasmids were sequenced, using a R.9.4.1 MinION Flow Cell (Oxford Nanopore Technologies, Oxford, United Kingdom) in accordance to previous work [9].

**Table 1:** Overview of the PPTases used in this study

PPTase	Origin	Size (aa)	Accession number
Gsp	*B. brevis*	242	CAA53988.1
Sfp	*B. subtilis *	224	WP_101501862.1
SpPPT1	*S. pombe*	258	SPAC17C9.02c
NpgA	*A. nidulans *	344	AN6140.2
FgPPT1	*F. graminearum*	309	FGSG_08779
FsPPT1	*F. solani*	315	NECHADRAFT_35672
FvPPT1	*F. verticillioides*	292	FVEG_01894

### Expression, purification and chemical analysis

Single colonies of each transformants were incubated in 5 mL appropriate selective drop-out medium containing (SC-LEU) 2% glucose over night at 30 ℃, 200 rpm. Overnight cultures were vortexed and cell density was estimated using a NanoDrop 2000c (Thermo Fisher Scientific, Waltham, Massachusetts, USA). The cells were diluted into a 250 mL baffled shake flasks containing 50 mL selective media with 2% raffinose [D(+)-raffinose pentahydrate, Acros organics, China] to an OD600  = 0.2. The cells were grown for approximately 6–8 h at 30 ℃, 200 rpm until an OD600 of 1 was achieved. Galactose [D(+)-galactose, VWR chemicals, Belgium] was added to a final concentration of 4% in order to induce transcription, by activating the GAL1/10 promoter system in the pESC-vector system. The cultures were maintained at 30℃ and 200 rpm for 48 h to induce expression of the fungal biosynthetic genes. After 48 h, cultures were pelleted and purification were performed on the pellet and supernatant independently, utilizing the methods described in previous work for the pellet and supernatant respectively [28, 29]. The dried extracts were dissolved in 1 mL methanol and analyzed on a Hitachi Elite LaChrom HPLC in accordance to chemical analysis performed in [30].

### Alignment analyses and 3D-modelling

To compare the ACP domains of Fsr1 and Bik1, the amino acid sequences were aligned in CLC Main Work Bench (CLC Bio-Qiagen, Aarhus, Denmark) CLC using the clustalW algorithm. Homology models of the three-dimensional structures, of the seven PPTases and the ACP domains of the two PKSs, Bik1 and Fsr1, were generated using the SWISS-MODEL modeling server [31–35]. ACP models were based on the structurally elucidated ACP domain from the norsolorinic acid synthase (NSAS), which is involved in aflatoxin biosynthesis in *Aspergillus parasiticus* (PDB accession number: 2KR5) [36]. The PPTases were modelled using the crystal structure of Sfp (PDB accession number: 4MRT) [37]. These templates were chosen since they had the highest sequence identity to the PPTases and ACP domains included in our study. The results were subsequently visualized with icn3d viewer [38, 39].

The chosen PPTases were subjected to a phylogenetic analysis to visualize their evolutionary relationship. This analysis included 22 additional published PPTases, which had previously been used in a similar study [40].^1^

## Results and discussion

### Tertiary structure comparison of the ACP domains and PPTases

**Fig. 3:**
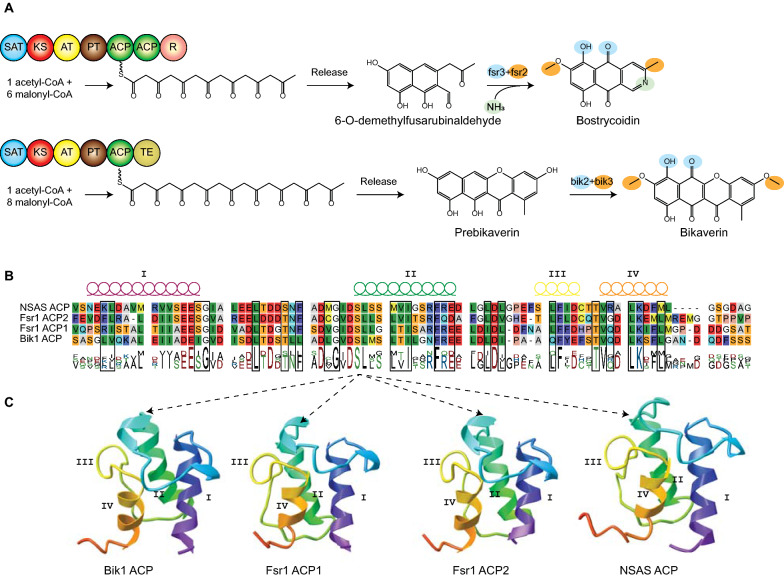
**A** A simplistic visualization of the two biosynthetic pathways of bikaverin and bostrycoidin, spanning the release from the ACP-domain to completed synthesis by the subsequent tailoring enzymes. **B** Multiple alignment of the three ACP-domains in Bik1 and Fsr1, alongside the template ACP-domain of NSAS used for structure prediction. The predicted α-helix structures are denoted on top of the alignment **C** 3D-structure of the ACP-domains native to Bik1 and Fsr1, showing the structural similarity of the four ACP-domains despite the sequence variation. The serine residue of the DxGxDSL motif where the phosphopantetheinyl arm is attached by the PPTase is highlighted

The biosynthetic pathways for bikaverin and bostrycoidin share several similarities. Both are initiated by a non-reducing PKS, Bik1 and Fsr1, which recruits eight and six malonyl-CoA, respectively, in addition to one acetyl-CoA (Fig. 3A). The resulting products, 6-O-demethylfusarubinaldehyde and prebikaverin are both subsequently subjected to oxygenation (Bik2 and Fsr3) and O-methylation (Bik3 and Fsr2) to yield the final quinone pigments. The two most noticeable differences between the two PKSs are that Fsr1 contains two ACP domains and a reductase domain for product release, while bik1 has a single ACP domain and uses a thioesterase for product release. Initial pairwise analyses showed that the two ACP domains in Fsr1 share 50% sequence identity on amino acid level. Furthermore, the Bik1 ACP domain had 56% and 44% sequence identity to Fsr1 ACP1 and ACP2, respectively. Three-dimensional modelling of the Bik1 and Fsr1 ACP domains showed strong similarities to the structurally elucidated NSAS ACP from *Aspergillus parasiticus*, which contain four helices (Fig. 3B, C). Three of these helices (I, II and IV) are arranged in parallel, while the short helix III is more rigid due to hydrophobic packing amino acids [36]. The sequence analyses showed that many of the restricting motives are present in the Bik1 and Fsr1 ACP domains, including the DxGxDSL motif where the phosphopantetheinyl arm is attached by the PPTases [41].^2^ This suggests that they may have variable affinity to substrates and protein–protein interaction with PPTases.

To determine the structural differences of the seven selected PPTases, they were also subjected to sequence analyses and three-dimensional modeling. Phylogenetic analysis of the PPTases showed clustering patterns according to the evolutionary origin, where the bacterial PPTases (sfg and GSP) are located in one clade, while the fungal PPTases are located in a separate clade (Additional file 1: Figure S1).

**Fig. 4:**
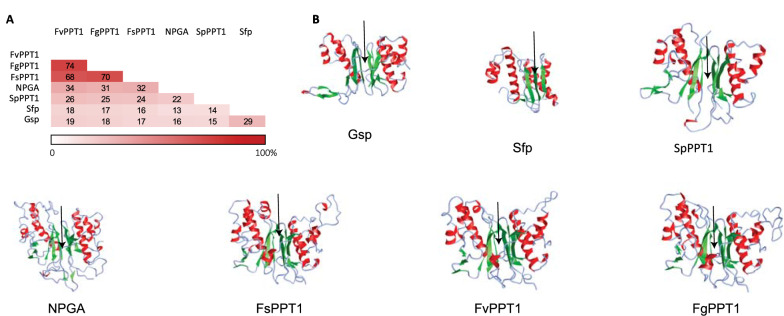
**A** Similarity matrix comparing the seven PPTases investigated regarding sequence identity. **B** Predicted 3D-structures of the PPTases, illustrating the heterogeneity in both secondary and overall tertiary structure between the investigated PPTases. The arrows indicate the sites for interaction with the ACP domains

The peptide sequence identity ranged from 13 to 74%, with Sfp from *Bacillus subtilis* being the most distantly related (Fig. 4A). The differences reflected the biological origin, as the three *Fusarium* PPTases were 68–74% identical. These were however only 31–34% identical to the *A. nidulans* NpgA and 22–26% to SpPPT1 from *S. pombe*. The structurally elucidated Sfp displayed a pseudo two-fold symmetry with two halves of equal sizes as previously described [42] (Additional file 1: Figure S2). Despite the difference in protein size and sequence, similar fold patterns were observed for all the selected PPTases, including the sites for interaction with the ACP domains (Fig. 4B). The larger fungal PPTases did however contain larger flexible regions compared to the two bacterial PPTases Sfp and Gsp.

### Effect of PPTases on bikaverin production

**Fig. 5:**
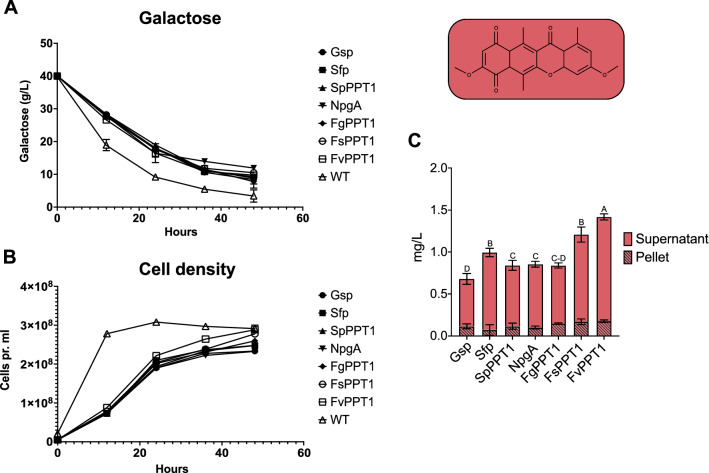
Growth experiments for production of bikaverin, where **A** illustrates the consumption of galactose every 12 h throughout the experiment by respective strains. **B** Growth rate of the individual bikaverin producing strains, compared to the wild-type (WT) *S. cerevisiae* BY4743. **C** Production levels of bikaverin in the individual strains, accumulated in both the pellet and supernatant

The individual PPTases were partnered with the two different biosynthetic pathways, resulting in the production of the two polyketides bikaverin and bostrycoidin. A library of each pathway and PPTase combination was isolated and screened for production, before biological triplicates were inoculated for production. To monitor differences in growth patterns, the growth of the yeast strains and consumption of galactose was measured every 12 h throughout the experiment. The results revealed a significantly lower galactose consumption rate for the expression strains compared to the WT-strain (Fig. 5A). Furthermore, all expression strains displayed a slower growth rate than the WT-strain (Fig. 5B), which correlates with the consumption results and can be explained by the plasmid burden and metabolic load of bikaverin production. The strain expressing *FvPPT1* exhibited the highest growth rate of the expression strains, however not significantly higher than the remaining. After 48 h of induction, the cultures were centrifuged and bikaverin concentrations were determined in pellets and supernatants. Overall, the seven individual PPTases displayed their ambiguous nature and successfully transferred the phosphopantetheinyl moiety to the polyketide synthase Bik1, resulting in the production of prebikaverin and subsequently bikaverin, yielding concentrations ranging from 0.7 to 1.4 mg/L. These results are similar to previously observed galactose induced bikaverin production in *S. cerevisiae* of 0.6 mg/L with NpgA as the assisting PPTase [11]. A small amount, 0.1–0.18 mg/L, was also found in the pellet of each culture, however non exhibited significantly higher or lower levels of accumulated bikaverin when compared to the other strains. The most prolific producer of bikaverin was the *FvPPT1-*strain, which yielded around 1.4 mg/L (Fig. 5C), a significantly higher level than all other strains, proven by performing one-way ANOVA (*p*  < 0.05) on the measured production levels. The *FsPPT1* and *sfp-*strain also showed significantly higher production than the remaining strains outside of *FvPPT1.* Although this increased yield could be partly be due to the slightly higher growth rate of the *FvPPT1-*strain (Fig. 5B), the match between PKS and PPTase could simply be more efficient and therefore cause the highest production level. These results were also observed when comparing the production relatively to the growth (Additional file 1: Figure S3). This would indicate an evolutionary advantage, that partnering Bik1 and FvPPT1 which both originate from *F. verticillioides*, could the reason for the highest production level. However, other factors, such as protein stability, may contribute to the observed results.

### Effect of PPTases on bostrycoidin production

**Fig. 6:**
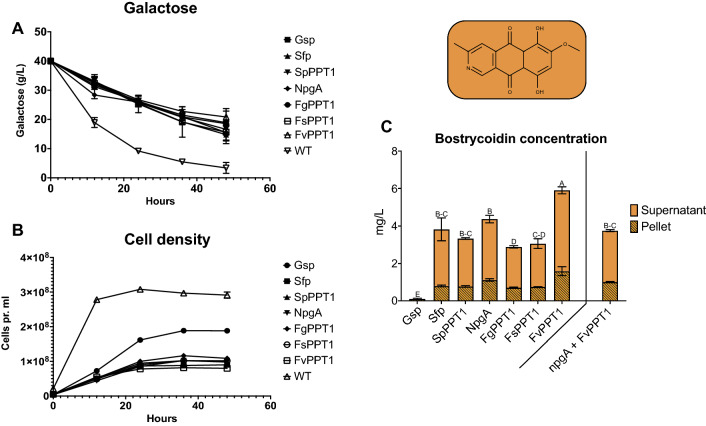
Growth experiments for production of bostrycoidin, where **A** illustrates the consumption of galactose every 12 h throughout the experiment. **B** Growth rate of the individual bostrycoidin producing strains, compared to the wild-type (WT) *S. cerevisiae* BY4743. **C** Production levels of bostrycoidin in the individual strains, accumulated in both the pellet and supernatant. Furthermore, production levels of bostrycoidin from the additional expression strain containing two PPTase-genes (*npgA* and *FvPPT1*) is included, adjacent to the initial growth experiments

In the second case study with bostrycoidin, we followed the same experimental setup as for bikaverin. Again, we observed that the galactose consumption rates were similar for the all expression strains, whereas the WT-strain consumed the most throughout the experiment (Fig. 6A). The faster consumption of galactose in medium incubated with the WT-strain was also reflected in a higher accumulation of cells (Fig. 6B). Interestingly, the *gsp-*strain grew significantly faster than the other expression strains, which most likely is explained by its inability to produce bostrycoidin in detectable levels (Fig. 6C). This suggests that the protein–protein interaction between the ACP-domains in Fsr1 and the PPTase Gsp*,* is insufficient for transferring the moiety, thus not altering the ACP-domains towards the functional holo-structure, leading to almost no production of bostrycoidin. The two ACP-domains in Fsr1 only exhibit around 50% sequence variations from the ACP-domain of Bik1, which could be a factor resulting in the apparent unsuccessful interaction, as Gsp was able to phosphopantheteinylate Bik1, but not Fsr1. The combination of Bik1 and Gsp also yielded the lowest concentration, which could indicate that this bacterial PPTase is less efficient at interacting with fungal PKS. The remaining PPTases could all support bostrycoidin production, with levels ranging from 3.5 to 5.9 mg/L. Here, as well as observed during the production of bikaverin, the co-expression of *FvPPT1* yielded the highest polyketide production at 5.9 mg/L, a production significantly higher than the remaining strains. The bostrycoidin accumulated in the pellet was simultaneously significantly higher for the *FvPPT1-*strain, than the remaining strains. Likewise, the *sfp-*strain also showed high levels of production, alongside the *npgA-*strain, which both exhibited significantly higher levels of bostrycoidin than the remaining strains.

The FvPPT1 enzyme thereby exhibited the highest efficiency in both systems as it accumulated more of the target polyketides in both pellets and supernatant. A broader study containing a plethora of other pathways from different origins would indicate whether this is a universally optimal PPTase partner or if it is only highly efficient when working with the *Fusarium* PKSs, as in this study. In this regard, it is worth noticing that *F. verticillioides* also contains the functional Fsr-gene cluster homologous to the *fsr-genes* expressed in the bostrycoidin pathway [43, 44], and therefore has followed the same evolutionary path.

The results of the bikaverin and bostrycoidin production (Figs. 5C,  6C and Additional file 1: Figure S3) revealed that heterologous expression of polyketides can be optimized by matching a polyketide synthase of interest, with the optimal PPTase-partner, instead of utilizing the more commonly applied NpgA, GSP and Sfp [10, 17, 45]. Furthermore, we wanted to elucidate the potential stacking effect of PPTases, the two most efficient PPTases (NpgA and FvPPT1) were co-expressed in a single organism to produce bostrycoidin through the introduction of a third plasmid. Adding multiple PPTases has been shown to further increase production of target polyketides [11], however in this case (Fig. 6C) the bostrycoidin production was affected negatively. This could be correlated to the increased plasmid burden of the vector-system utilized, which carry a high-copy number origin [46].

### Conclusion

The two target polyketides were successfully expressed in *S. cerevisiae* alongside the seven PPTases. Of the 14 PKS and PPTase combinations, 13 showed successful interactions resulting in production of the target metabolite and one resulting in miniscule production of target polyketide. The expression of bostrycoidin and bikaverin both resulted in higher extracellular concentration of target polyketides, than what accumulated in the pellet. The study furthermore showed that FvPPT1 from *F. verticillioides* was the most efficient PPTase partner for heterologously expressing these two biosynthetic pathway in *S. cerevisiae* and the production of PKS can thus be increased through utilization of pathway-specific PPTases. Most importantly, the study elucidated that the optimal combination of PKS and PPTase can increase production of target polyketide up to two-fold, compared to the most widely applied PPTases in heterologous expression systems.

## Supplementary Information

**Additional file 1: Table** **S1. **This table contains the primer sequences of both the primers used for gene-amplification and the primer used for initial sanger-sequencing in fragments of around 700 bp, containing at least 50 bp overlap between each fragment. **Table S2. **This table contains the different plasmids utilized in the project, both the native plasmids used as expression vectors, but also plasmids purchased containing the synthetically derived codon optimized genes. **Figure S1. **Phylogenetic tree of the PPTases used in the present study (bold) together with 22 additional published PPTases. Bootstrap values (> 70%) from 1000 replications are indicated at the respective nodes. **Figure S2. **Predicted structure of sfp/ACP interaction with the CoA and Mg^2+^ ion highlighted by arrows. **Figure S3. **Production levels of bikaverin and bostrycoidin in the individual strains (relative to OD at 48 h) in the supernatant and pellets. The mean of the supernatant from BY4743::*FvPPT1 *was set to 100 for both compounds.

## Data Availability

The plasmids and datasets used and/or analyzed during the current study are available from the corresponding author on reasonable request.

## Footnotes

1 https://www.ncbi.nlm.nih.gov/pmc/articles/PMC3918677/.

2 https://pubs.rsc.org/en/content/articlelanding/2012/NP/c2np20019h.
