# Supplementary material for: Speed dating for enzymes! Finding the perfect phosphopantetheinyl transferase partner for your polyketide synthase
Source: Microb Cell Fact. 2022 Jan 10;21:9. doi: 10.1186/s12934-021-01734-9 (PMC8751348; doi:10.1186/s12934-021-01734-9)

# Additional file 1

**Table S1.** This table contains the primer sequences of both the primers used for gene-amplification and the primer used for initial sanger-sequencing in fragments of around 700 bp, containing at least 50 bp overlap between each fragment.

| Gene | Fragments | Product size (bp) | Name | TAR-region and annealing sequence for PCR |
|---|---|---|---|---|
| fsr1 | 2 | 3274 | Fsr1.1-fw | AAA ATT CGA ATT CAA CCC TCA CTA AAG GGC<br>ATG ACA GAC AAC TTA AAA TTA TAC TTA TTC G |
| | | | Fsr1.1-rv | CCT TCA AAG CTG CAC ACA AA |
| | | 3291 | Fsr1.2-fw | TTC CAT ACG CAT TCC ATT CA |
| | | | Fsr1.2-rv | ACA ACC TTG ATT GGA GAC TTG ACC AAA CCT<br>TCA AAC TCT TGG ACC CCA CA |
| fsr2 | 2 | 563 | Fsr2.1-fw | ATA CTT TAA CGT CAA GGA GAA AAA ACC CCG<br>ATG CAC AAG ACT GAA AGA GAC G |
| | | | Fsr2.1-rv | GGT GGC GGT AGA ACC GCT GCT TCC ACC AAC<br>ATC AAC GAC CTT GGC CTC T |
| | | 618 | Fsr2.2-fw | AAG GCT CTG GGA GAG GCC AAG GTC GTT GAT G<br>TTG GTG GAA GCA GCG GT |
| | | | Fsr2.2-rv | GAT CTT AGC TAG CCG CGG TAC CAA GCT TAC<br>CTA AGC ATG CCC ATT CAG ACC |
| fsr3 | 1 | 1599 | Fsr3-fw | AAA ATT CGA ATT CAA CCC TCA CTA AAG GGC<br>ATG CAA ATC AAC GAC CAA AC |
| | | | Fsr3-rv | GCC GAC AAC CTT GAT TGG AGA CTT GAC CAA<br>CTA TGC CCA GTC ACC GTC TT |
| bik1 | 1 | 6111 | Bik1.Fw | AAA ATT CGA ATT CAA CCC TCA CTA AAG GGC GGC C<br>ATG GCC TCC TCC GCA GAT GT |
| | | | Bik1.Rv | TCT GGC GAA GAA TTG TTA ATT AAG AGC TCA<br>TCA GTT GAC ACC CAT TGC TT |
| bik2 | 1 | 1470 | Bik2.Fw | AAA ATT CGA ATT CAA CCC TCA CTA AAG GGC GGC C<br>ATG GCT GAA CCA AAC CAA CA |
| | | | Bik2.Rv | TCT GGC GAA GAA TTG TTA ATT AAG AGC TCA<br>TTA AGA ACC AAC TTC AAC AAC ACC |
| bik3 | 1 | 1362 | Bik3.Fw | ATA CTT TAA CGT CAA GGA GAA AAA ACC CCG<br>ATG GTT TCT AAC GGT ATC TCA |
| | | | Bik3.Rv | GCG GAT CTT AGC TAG CCG CGG TAC CAA GCT<br>TTA ACC TAA AAC AAC ATC AAT AAC TGA C |
| npgA | 1 | 1035 | npgA.Fw | ATA CTT TAA CGT CAA GGA GAA AAA ACC CCG<br>ATG GTT CAA GAT ACT TCT TCA GCT T |
| | | | npgA.Rv | GAT CTT AGC TAG CCG CGG TAC CAA GCT TAC<br>TTA AGA TAA ACA ATT ACA AAC ACC TGT AGC |
| gsp | 1 | 729 | gsp.Fw | ATA CTT TAA CGT CAA GGA GAA AAA ACC CCG<br>ATG GGT GGT CAA AAG ATG AT |
| | | | gsp.Rv | GAT CTT AGC TAG CCG CGG TAC CAA GCT TAC<br>TTA AAA ATT ATT ATT TTC TGA AAA AGT AGA |
| sfp | 1 | 672 | sfp.Fw | ATA CTT TAA CGT CAA GGA GAA AAA ACC CCG<br>ATG AAA ATC TAC GGT ATC TAC ATG GA |
| | | | sfp.Rv | GAT CTT AGC TAG CCG CGG TAC CAA GCT TAC<br>TAA CAA TTC TTC GTA GGA AAC CAT AG |
| FgPPT | 1 | 930 | FgPPT.Fw | ATA CTT TAA CGT CAA GGA GAA AAA ACC CCG<br>ATG TCT CAA ACT CAA TCT TCA CCA |
| | | | FgPPT.Rv | GAT CTT AGC TAG CCG CGG TAC CAA GCT TAC<br>TTA TGA AGA TGG CAA TCT TTC ACC |
| FsPPT | 1 | 948 | FsPPT.Fw | ATA CTT TAA CGT CAA GGA GAA AAA ACC CCG<br>ATG GGT GAA TCT ACT CCA ACA G |
| | | | FsPPT.Rv | GAT CTT AGC TAG CCG CGG TAC CAA GCT TAC<br>TTA TAA AGC ATC TGT TGC ATC TTC |
| q10474 | 1 | 780 | q10474.Fw | ATA CTT TAA CGT CAA GGA GAA AAA ACC CCG<br>ATG AAG CAA AAG GTT TAC AGA TTG T |
| | | | q10474.Rv | GAT CTT AGC TAG CCG CGG TAC CAA GCT TAC<br>TTA CAA ATC ATT CAA TGT TTC CCA |
| FvPPT | 1 | 879 | FvPPT-fw | ATA CTT TAA CGT CAA GGA GAA AAA ACC CCG<br>ATG TCC TCA GCA CAA TCA TCA |
| | | | FvPPT-rv | GAT CTT AGC TAG CCG CGG TAC CAA GCT TAC<br>TTA TGA TTT AGG AGC CTT TTC ACC |

**Table S2.** This table contains the different plasmids utilized in the project, both the native plasmids used as expression vectors, but also plasmids purchased containing the synthetically derived codon optimized genes.

| Plasmid | Gene inserted | Purchased | Constructed | Restriction enzymes for linearization | Resulting plasmid |
|---|---|---|---|---|---|
| pESC-URA | *empty* | • | | | |
| pESC-LEU | *empty* | • | | | |
| pUC57 | *fsr1* | • | | | |
| pUC57 | *fsr3* | • | | | |
| pUC57 | *bik1* | • | | | |
| pUC57 | *bik2* | • | | | |
| pUC57 | *bik3* | • | | | |
| pUC57 | *npgA* | • | | | |
| pUC57 | *gsp* | • | | | |
| pUC57 | *sfp* | • | | | |
| pUC57 | *FgPPT* | • | | | |
| pUC57 | *FsPPT* | • | | | |
| pJET1.2 | *Q10474* | • | | | |
| pUC57 | *FvPPT* | • | | | |
| pESC-LEU | *fsr1* | | • | NotI/BglII | pESC-LEU::*fsr1* |
| pESC-LEU+*fsr1* | *npgA* | | • | BamHI/XhoI | pESC-LEU::*npgA+fsr1* |
| pESC-LEU+*fsr1* | *gsp* | | • | BamHI/XhoI | pESC-LEU::*gsp+fsr1* |
| pESC-LEU+*fsr1* | *sfp* | | • | BamHI/XhoI | pESC-LEU:: *sfp +fsr1* |
| pESC-LEU+*fsr1* | *FgPpt* | | • | BamHI/XhoI | pESC-LEU::*FgPpt+fsr1* |
| pESC-LEU+*fsr1* | *FsPpt* | | • | BamHI/XhoI | pESC-LEU::*FsPpt+fsr1* |
| pESC-LEU+*fsr1* | *Q10474* | | • | BamHI/XhoI | pESC-LEU::*q10474+fsr1* |
| pESC-LEU+*fsr1* | *FvPpt* | | • | BamHI/XhoI | pESC-LEU::*FvPPT+fsr1* |
| pESC-URA | *fsr3* | | • | NotI/BglII | pESC-URA::*fsr3* |
| pESC-URA+*fsr3* | *fsr2* | | • | BamHI/XhoI | pESC-URA::*fsr2+3* |
| pESC-LEU | *bik1* | | • | NotI/BglII | pESC-LEU::*bik1* |
| pESC-LEU+*bik1* | *npgA* | | • | BamHI/XhoI | pESC-LEU::*npgA+bik1* |
| pESC-LEU+*bik1* | *gsp* | | • | BamHI/XhoI | pESC-LEU::*gsp+bik1* |
| pESC-LEU+*bik1* | *sfp* | | • | BamHI/XhoI | pESC-LEU:: *sfp +bik1* |
| pESC-LEU+*bik1* | *FgPpt* | | • | BamHI/XhoI | pESC-LEU::*FgPpt+bik1* |
| pESC-LEU+*bik1* | *FsPpt* | | • | BamHI/XhoI | pESC-LEU::*FsPpt+bik1* |
| pESC-LEU+*bik1* | *Q10474* | | • | BamHI/XhoI | pESC-LEU::*q10474+bik1* |
| pESC-LEU+*bik1* | *FvPpt* | | • | BamHI/XhoI | pESC-LEU::*FvPPT+bik1* |
| pESC-URA | *bik2* | | • | NotI/BglII | pESC-URA::*bik2* |
| pESC-URA+*bik3* | *bik3* | | • | BamHI/XhoI | pESC-URA::*bik2+3* |

**Figure S1.** Phylogenetic tree of the PPTases used in the present study (**bold**) together with 22 additional published PPTases. Bootstrap values (>70%) from 1000 replications are indicated at the respective nodes.

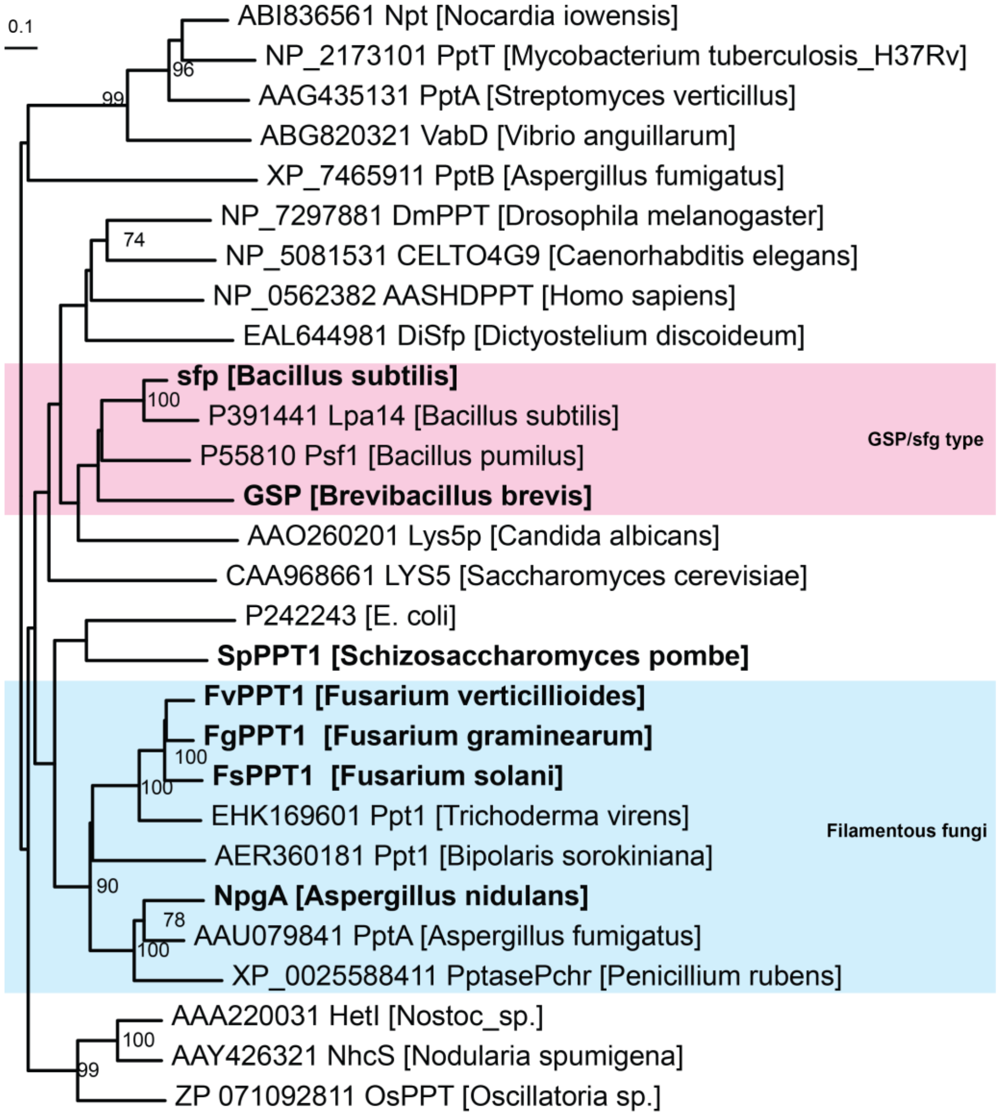

**Figure S2.** Predicted structure of sfp/ACP interaction with the CoA and Mg2+ ion highlighted by arrows.

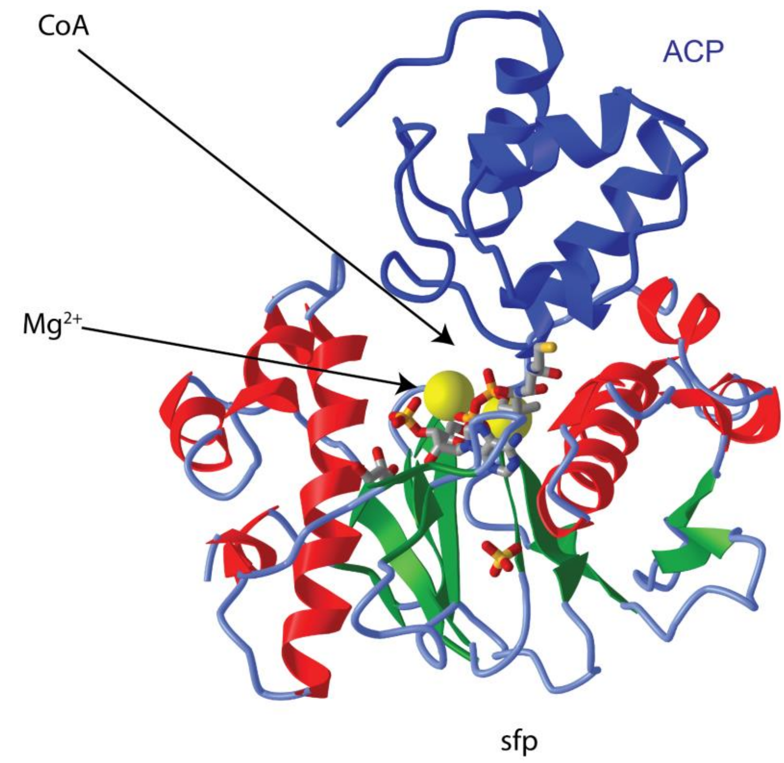

**Figure S3.** Production levels of bikaverin and bostrycoidin in the individual strains (relative to OD at 48 hours) in the supernatant and pellets. The mean of the supernatant from BY4743::*FvPPT1* was set to 100 for both compounds.

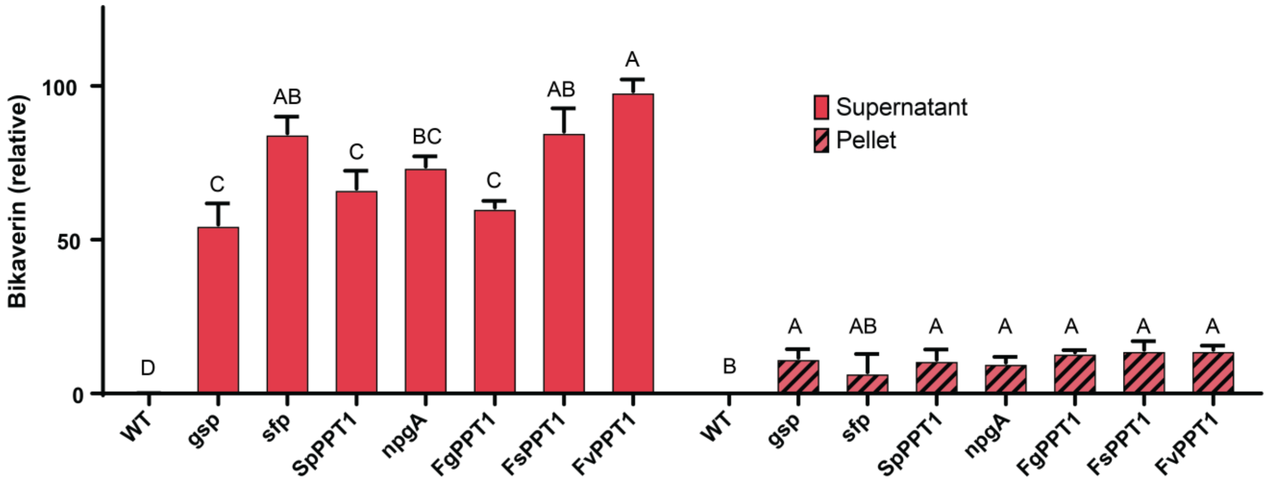


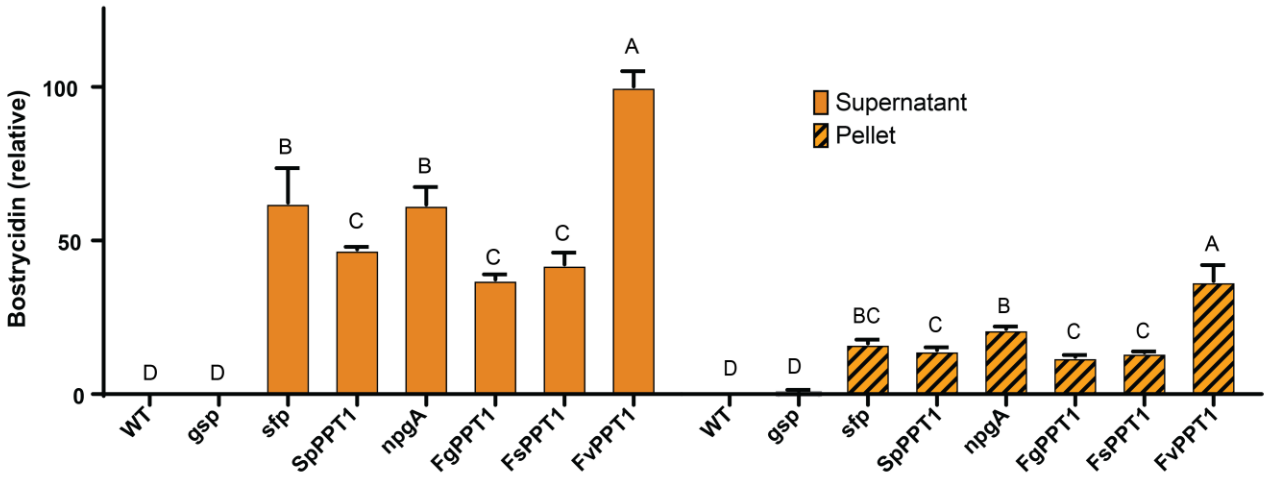

Supplement: Supplementary file 1 — Additional file 1: Table S1. This table contains the primer sequences of both the primers used for gene-amplification and the primer used for initial sanger-sequencing in fragments of around 700 bp, containing at least 50 bp overlap between each fragment. Table S2. This table contains the different plasmids utilized in the project, both the native plasmids used as expression vectors, but also plasmids purchased containing the synthetically derived codon optimized genes. Figure S1. Phylogenetic tree of the PPTases used in the present study (bold) together with 22 additional published PPTases. Bootstrap values (> 70%) from 1000 replications are indicated at the respective nodes. Figure S2. Predicted structure of sfp/ACP interaction with the CoA and Mg2+ ion highlighted by arrows. Figure S3. Production levels of bikaverin and bostrycoidin in the individual strains (relative to OD at 48 h) in the supernatant and pellets. The mean of the supernatant from BY4743::FvPPT1 was set to 100 for both compounds. [file 12934_2021_1734_MOESM1_ESM.pdf]
